# Supplementary material for: PH13 improves soybean shade traits and enhances yield for high-density planting at high latitudes
Source: Nat Commun. 2023 Oct 26;14:6813. doi: 10.1038/s41467-023-42608-5 (PMC10603158; doi:10.1038/s41467-023-42608-5)
Supplement: Supplementary file 1 — Supplementary Information [file 41467_2023_42608_MOESM1_ESM.pdf]

PH13 improves soybean shade traits and enhances yield for high-density planting at high latitudes

Qin et al.

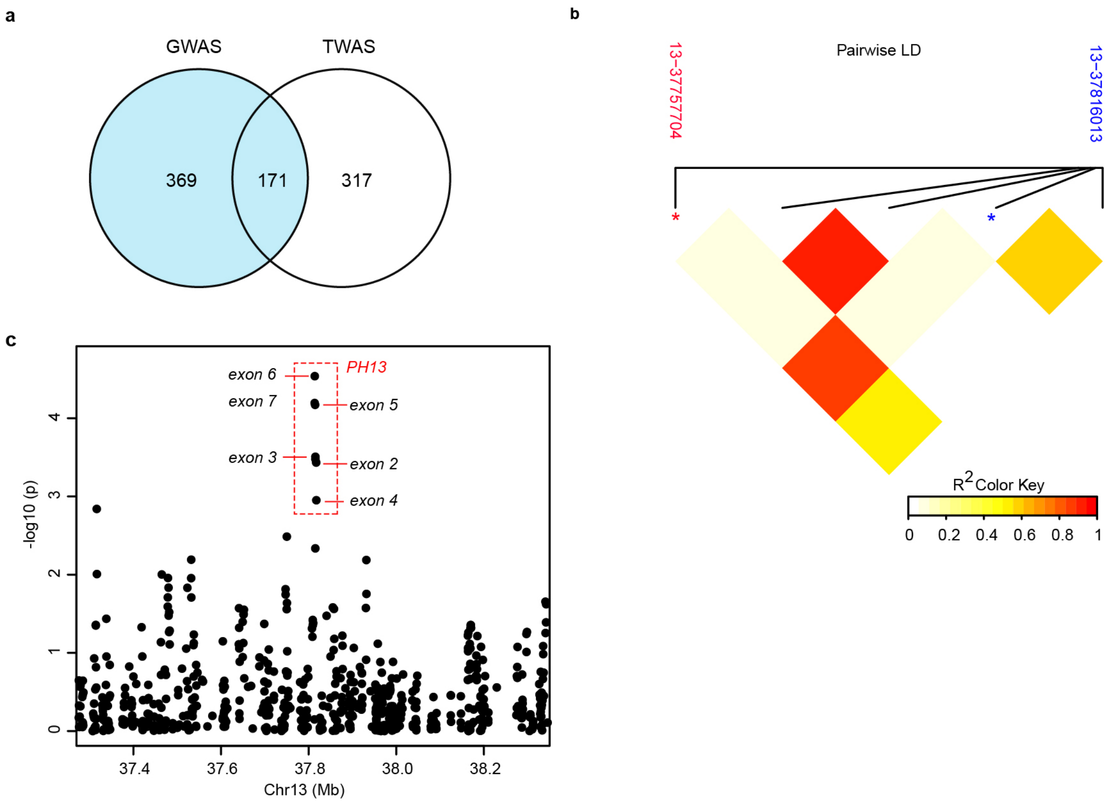

Supplementary Fig. 1: Identification of *PH13* as a candidate gene regulating plant height in soybean.

a, Sample number for GWAS and TWAS in this study are shown. **b,** Pair-wise linkage disequilibrium (R2) between the leading SNP (highlighted in red) in the *PH13* locus and GWAS input SNPs within *PH13* are shown. The asterisk represents the position of SNP. **c,** Plant height TWAS using compressed mixed linear model. A 1 Mb window centered on *PH13* is showed. Each dot represents one gene or exon.

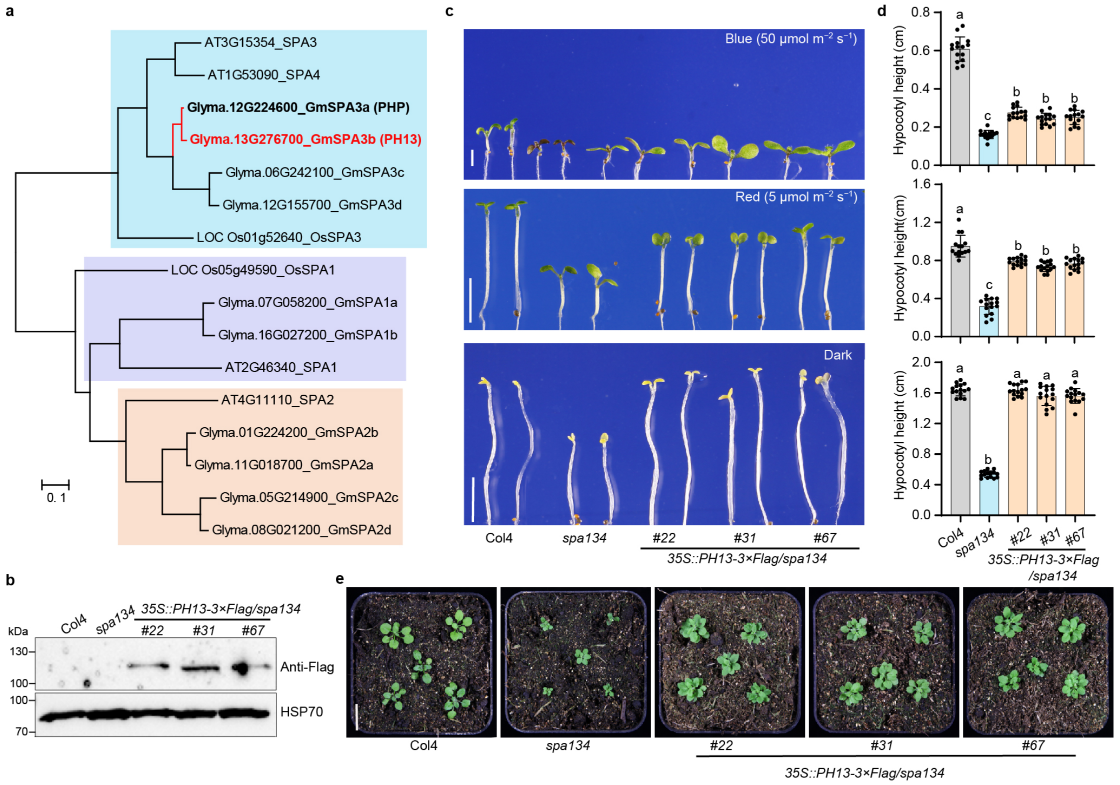

Supplementary Fig. 2: Ectopic expression of *PH13* in the *Arabidopsis spa134* mutant.

a, Phylogenetic analysis of SPA family members from *Arabidopsis thaliana*, rice, and soybean (Supplementary Data 4). The tree was constructed using the Maximum Likelihood method in the MEGA7 software. **b,** Immunoblot analysis of PH13-3×Flag protein expression in the indicated lines using the anti-Flag antibody. HSP70 protein was used as a loading control. **c,** Comparison of hypocotyl lengths of the indicated lines. Seedlings were grown for 4 days under blue light (50 μmol m^{-2} s^{-1}), red light (5 μmol m^{-2} s^{-1}), or darkness, respectively. Scale bar = 0.5 cm. **d,** Statistical analysis of hypocotyl length in (**c**). Data are means ± SD (n = 15 biologically independent plants). Lowercase letters above the dots indicate significant differences (one-way ANOVA with two-sided Tukey test at a significance level of 0.01). **e,** Visual phenotype of indicated plants grown under long day conditions in growth chamber for three weeks. Scale bar = 2 cm. Source data are provided as a Source Data file.

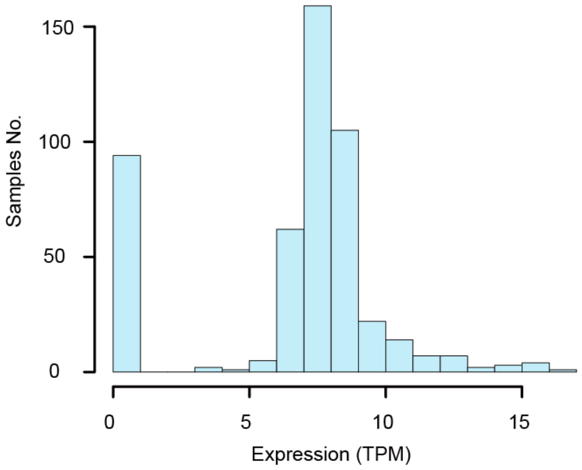

Supplementary Fig. 3: The expression variations at the 3' end of *PH13* identified by TWAS. Analysis of 488 accessions revealed two forms of *PH13* transcripts. The vertical axis depicts the number of samples (Samples NO.), while the horizontal axis depicts the expression levels (in TPM, Transcripts Per Kilobase Million).

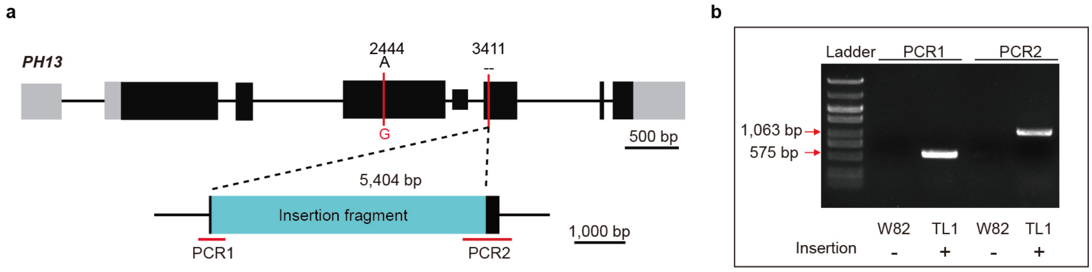

Supplementary Fig. 4: PCR genotyping for the insertion of *Ty1/Copia*-like retrotransposon in *PH13*^{H3}.

a, The genomic structure of the *PH13* gene (top) with the insertion fragment (bottom). **b,** PCR genotyping assay for the insertion fragment in TL1 cultivar harboring *PH13*^{H3}. The W82 cultivar carrying *PH13*^{H1} was used as a negative control. The 575 bp and 1,063 bp fragments represent the amplicons around left and right borders of the *Ty1/Copia*-like retrotransposon, respectively.

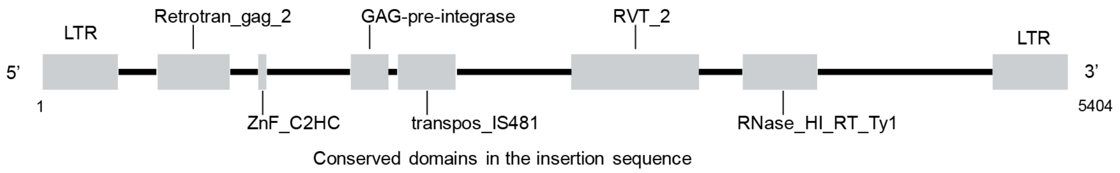

Supplementary Fig. 5: Analysis of the insertion sequence of the *Ty1/Copia*-like retrotransposon in *PH13*^{H3}.

The conserved domains of insertion sequence with a length of 5,404 bp were analyzed using the Blast N method in NCBI. A description of each domain in the insertion sequence is provided in Supplementary Data 5. The insertion sequence is listed in Supplementary Data 6.

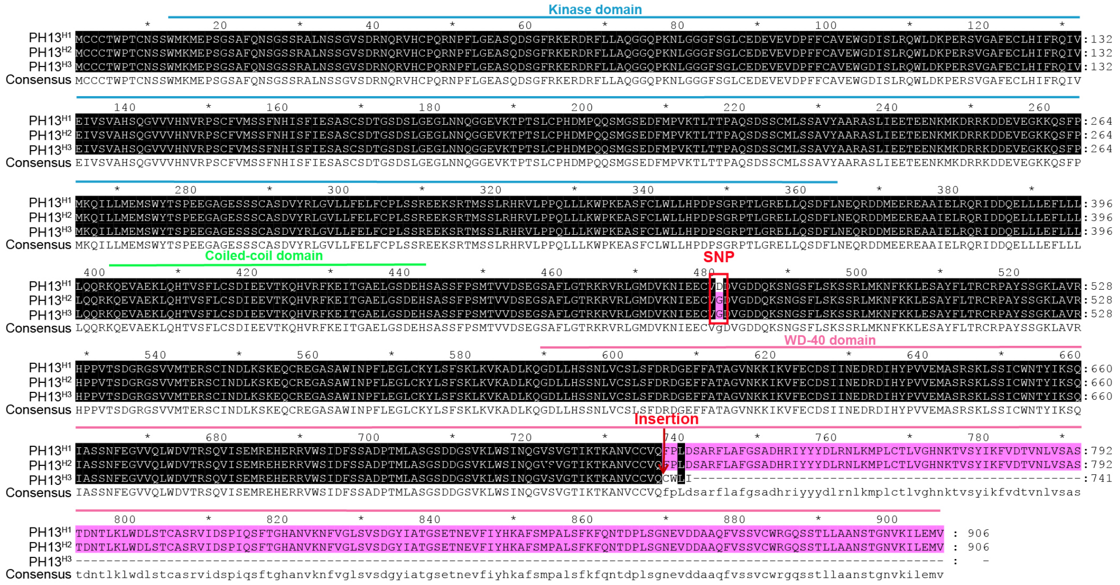

Supplementary Fig. 6: Alignment of amino-acid sequences of three major haplotypes of PH13.

The amino acid sequences were aligned by ClustalW Multiple alignments in MEGA7 and then manually adjusted using GeneDOC. The sequences that encode the Kinase domain are underlined in blue with the Coiled-coil domain denoted by the green line and the WD40 domain denoted by the pink line.

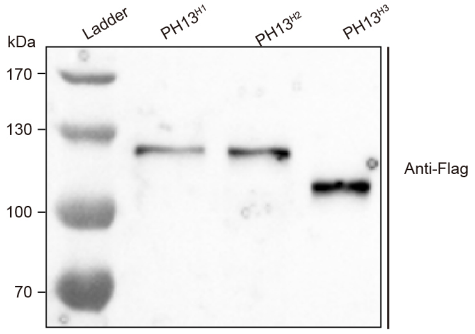

Supplementary Fig. 7: The protein expression level of each haplotype of *PH13* ectopically expressed in tobacco leaves.

The *35S::PH13*^{H1}*-3×Flag, 35S::PH13*^{H2}*-3×Flag*, and *35S::PH13*^{H3}*- 3×Flag* constructs were transformed into tobacco leaves, respectively. The transformed plants were incubated at 25℃ in dark for 12 hours and then grown under white light (WL, 80 μmol m^{-2} s^{-1}) for 36 hours. Total proteins were extracted from each sample, separated by SDS-PAGE gel and transferred onto a membrane. Target proteins were detected using an anti-Flag antibody (at a 1:2,500 dilution). The molecular weight of the protein product of each *PH13* haplotype was determined using a protein size marker (ladder).

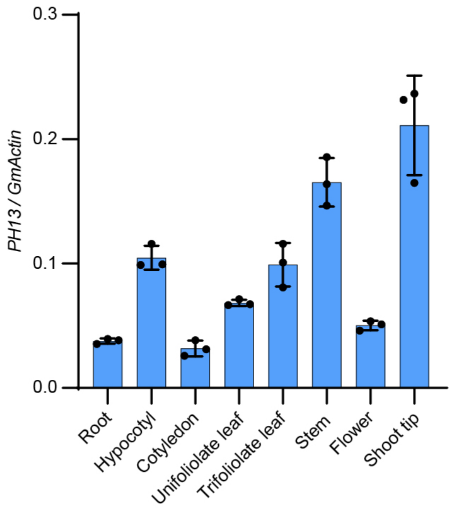

Supplementary Fig. 8: The tissue specific expression of *PH13*.

RT-qPCR analysis of *PH13* transcript levels in various tissues, including root, hypocotyl, cotyledon, unifoliate leaf, trifoliate leaves, stem, flower, and shoot tip of W82^{H1}. The relative expression level is shown as means ± SD (n = 3 biologically independent replicates). *GmActin* was used as the internal control. Source data are provided as a Source Data file.

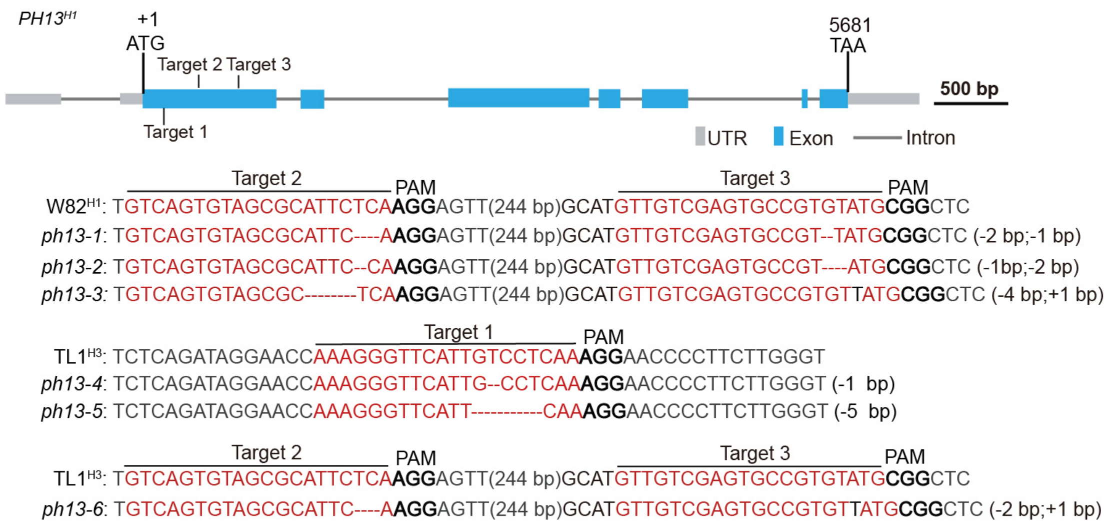

Supplementary Fig. 9: Generation of the CRISPR-Cas9 engineered *ph13* mutants.

Three independent single-guide RNAs were designed to target the exon 1 of *PH13*. The sequences of representative homozygous mutants (*ph13-1*, *ph13-2*, and *ph13-3*) in W82^{H1} and (*ph13-4*, *ph13-5*, and *ph13-6*) in TL1^{H3} background at the T2 generation are shown. The gRNA target sites are highlighted in red with the protospacer adjacent motif (PAM) in bold. Black letters and dashed lines within the target sites denote nucleotide insertion and deletion, respectively. Data of Sanger sequencing are provided as a Source Data file.

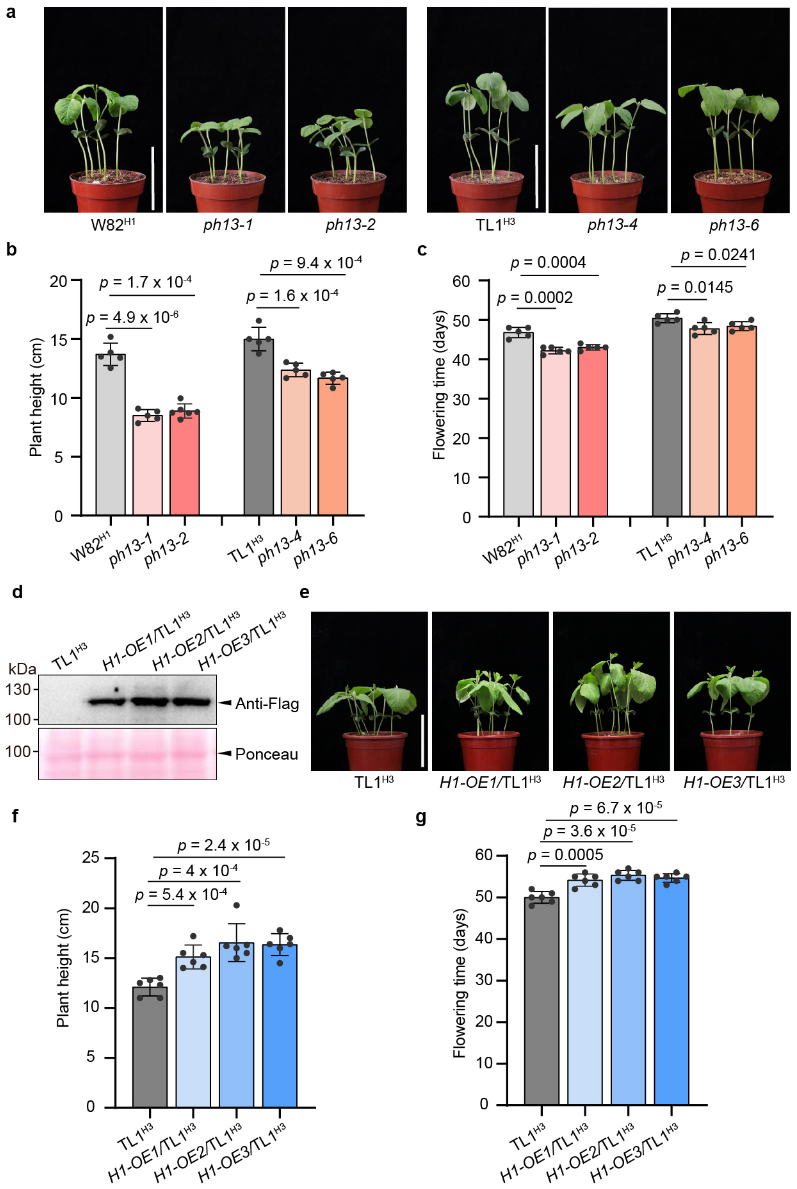

Supplementary Fig. 10: Phenotypic analysis of the *ph13* mutants and *PH13^{H1}* overexpression lines at the seedling stage.

a, Seedling photos of *ph13* mutants and WT plants grown under long day conditions (16 h light/8 h dark) in phytotron. **b and c,** Plant height and flowering time of indicated lines as in (**a**). Statistic data are shown as mean values ± SD (n = 5 biologically independent plants). **d,** Immunoblot showing the abundance of *PH13^{H1}-3×Flag* protein in the overexpression lines. **e,** Seedling photos of *PH13^{H1}* overexpression lines and WT TL1^{H3} grown under long day conditions. **f and g,** Plant height and flowering time of indicated lines as shown in (**e**). Statistic data are shown as mean values ± SD (n = 6 biologically independent plants). *p* values were calculated by unpaired, two-tailed Student's t-tests. Source data are provided as a Source Data file.

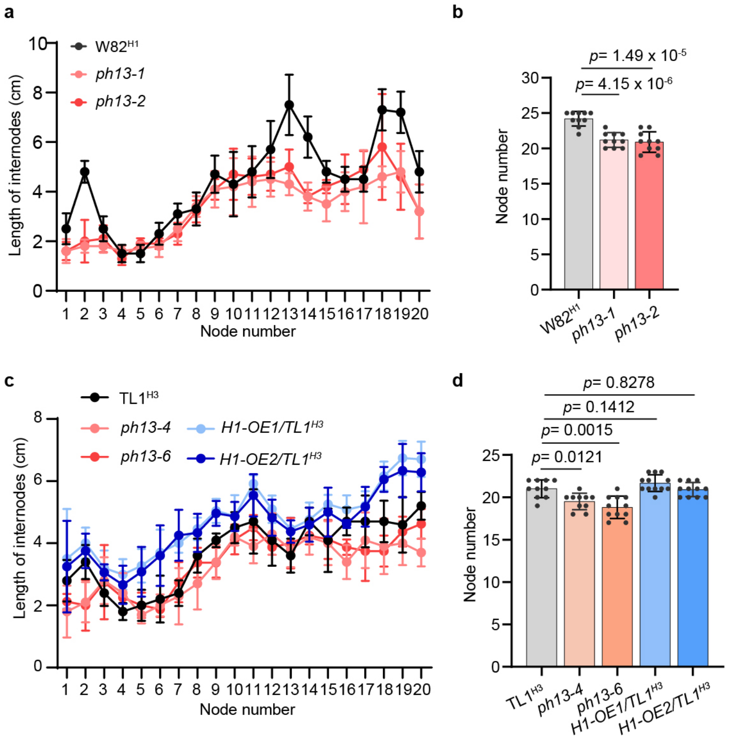

Supplementary Fig. 11: The effect of *PH13* on internode length and node number in soybean. **a-d,** Statistical analysis of internode length (**a and c**) and node number (**b and d**) of indicated lines. The above results were collected at R8 stage by field trials conducted in Beijing. Data are means ± SD (n = 5 biologically independent plants for **a, c** and n = 10 biologically independent plants for **b, d**) and p values were calculated by unpaired, two-tailed Student's t-tests. Source data are provided as a Source Data file.

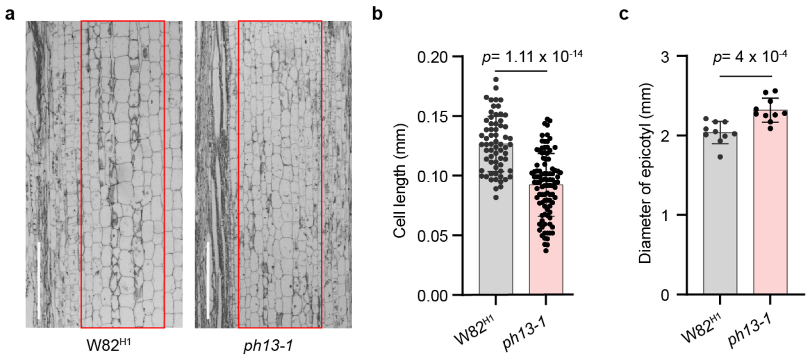

Supplementary Fig. 12: The cell morphology analysis of the *ph13-1* mutant and W82^{H1} at the seedling stage.

a, Epicotyl longitudinal sections of the WT W82^{H1} and *ph13-1* mutant at seedling stage. Scale bar, 0.5 mm. **b and c,** Measurements of xylem cell length (**b**, all the cells within each red rectangle were measured) and epicotyl diameter (**c**) of indicated lines as in (**a**). Statistic data are shown as mean values ± SD (n = 6 biologically independent plants, a representative one is shown for **a** and **b**). *p* values were calculated by unpaired, two-tailed Student's t-tests. Source data are provided as a Source Data file.

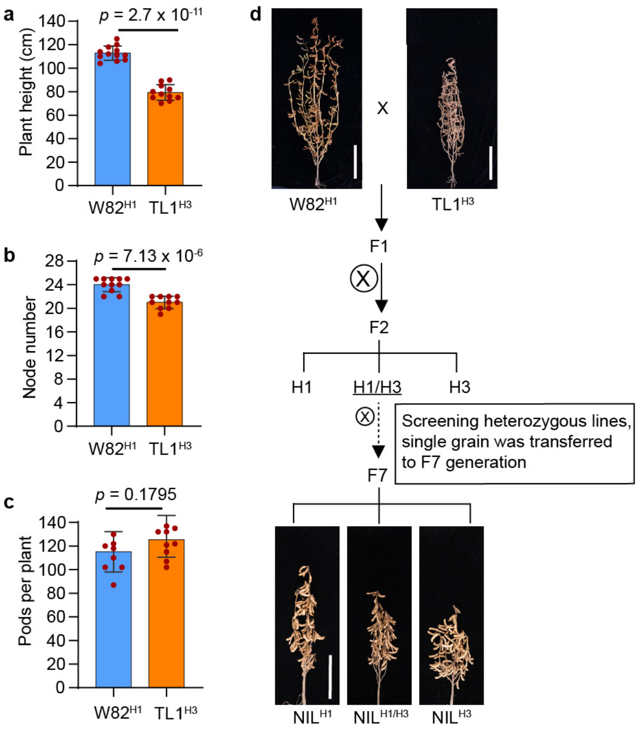

Supplementary Fig. 13: Generation of near-isogenic lines (NILs) differing at the *PH13* gene. **a-c,** Statistical analysis of the plant height (**a**), node number (**b**) and pod number (**c**) of W82^{H1} (carrying *PH13*^{H1}) and TL1^{H3} (carrying *PH13*^{H1}) cultivars. Data are shown as means ± SD (n = 10 biologically independent plants for **a-c**) with significant differences determined by two-tailed Student's t-test. **d,** Schematic illustration of the generation of NILs by crossing W82^{H1} with TL1^{H3}. A heterozygous line at the *PH13* gene was selected in the F7 generation progeny, and the individual segregating groups at F8 generation carrying homozygous H1 (NIL^{H1}) or homozygous H3 (NIL^{H3}) were used for phenotypic analysis. Phenotypic data were collected at R8 stage in Beijing field trails. Scale bar, 20 cm. Source data are provided as a Source Data file.

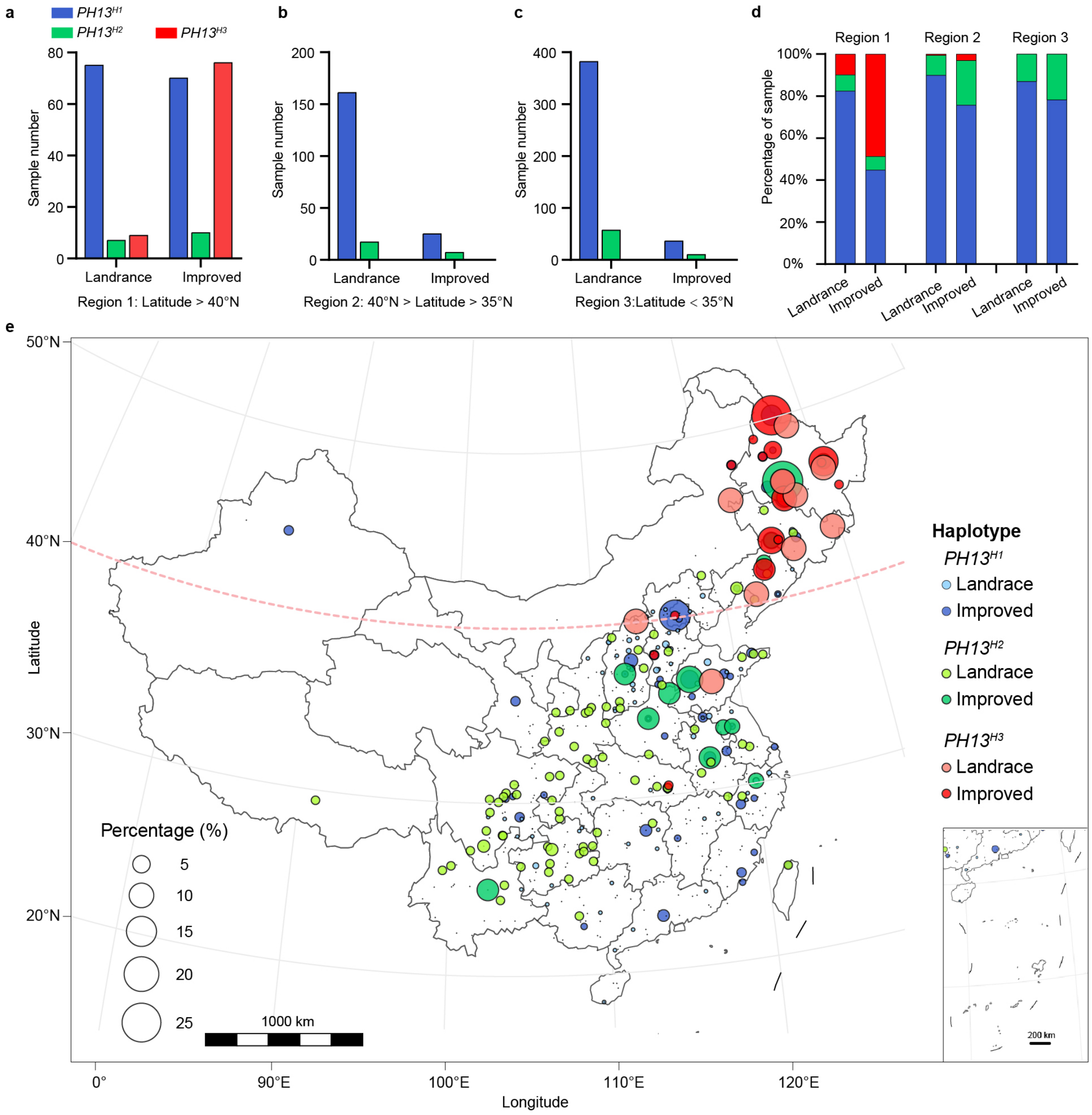

Supplementary Fig. 14: The geographical distribution of landrace and improved cultivars carrying *PH13*^{H1}, *PH13*^{H2}, and *PH13*^{H3} in China.

a-c, The sample number of *PH13* alleles (Latitude > 40°N, northern China) in Region 1 (**a**), Region 2 (40°N > Latitude > 35°N, central China surrounding the middle and low regions of Yellow River Valley) (**b**) and Region 3 (Latitude < 35°N, southern China) (**c**) of China. **d,** The haplotype frequency distribution of *PH13* in Region 1, Region 2, and Region 3 of China. **e,** The geographical distribution of 946 soybean accessions carrying *PH13*^{H1}, *PH13*^{H2}, or *PH13*^{H3} in China. Accession details are provided in Supplementary Data 7**.** The color and size of the circle represents the type and percentage of germplasm, respectively, in respective location.

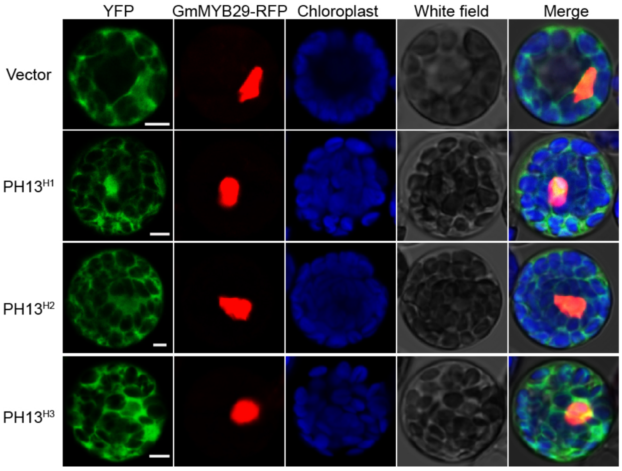

Supplementary Fig. 15: Subcellular localization of the three PH13 haplotypes.

Each construct of the indicated PH13 haplotype fused with YFP was ectopically expressed in *Arabidopsis* mesophyll protoplasts. The GmMYB29-RFP fusion protein was used as a nucleus marker. YFP alone was used as a negative control. Scale bar, 5 μm.

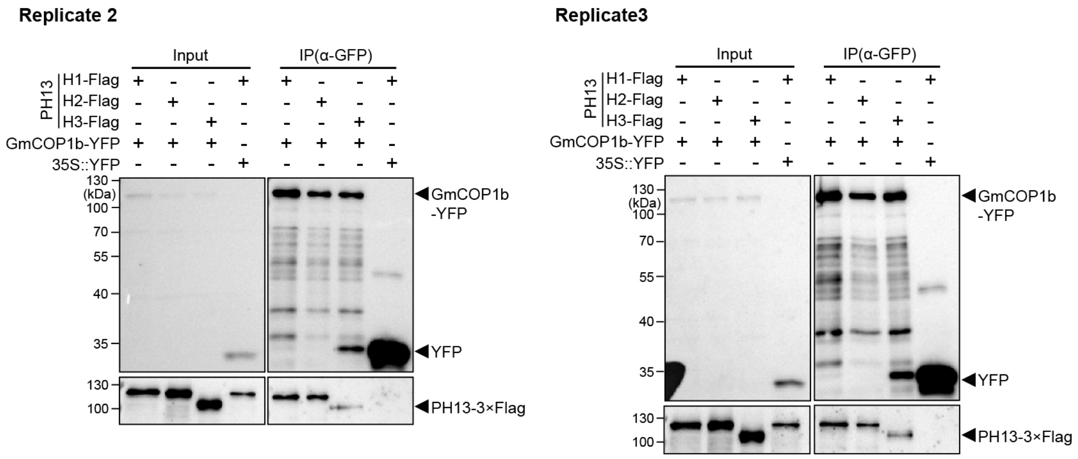

Supplementary Fig. 16: Analysis of interaction between each PH13 haplotype with GmCOP1b.

Biological replicate 2 and 3 of the Co-Immunoprecipitation (Co-IP) assay showing the interaction between each PH13 haplotype with GmCOP1b in tobacco leaves. Biological replicate 1 is shown in Fig. 4f. Source data are provided as a Source Data file.

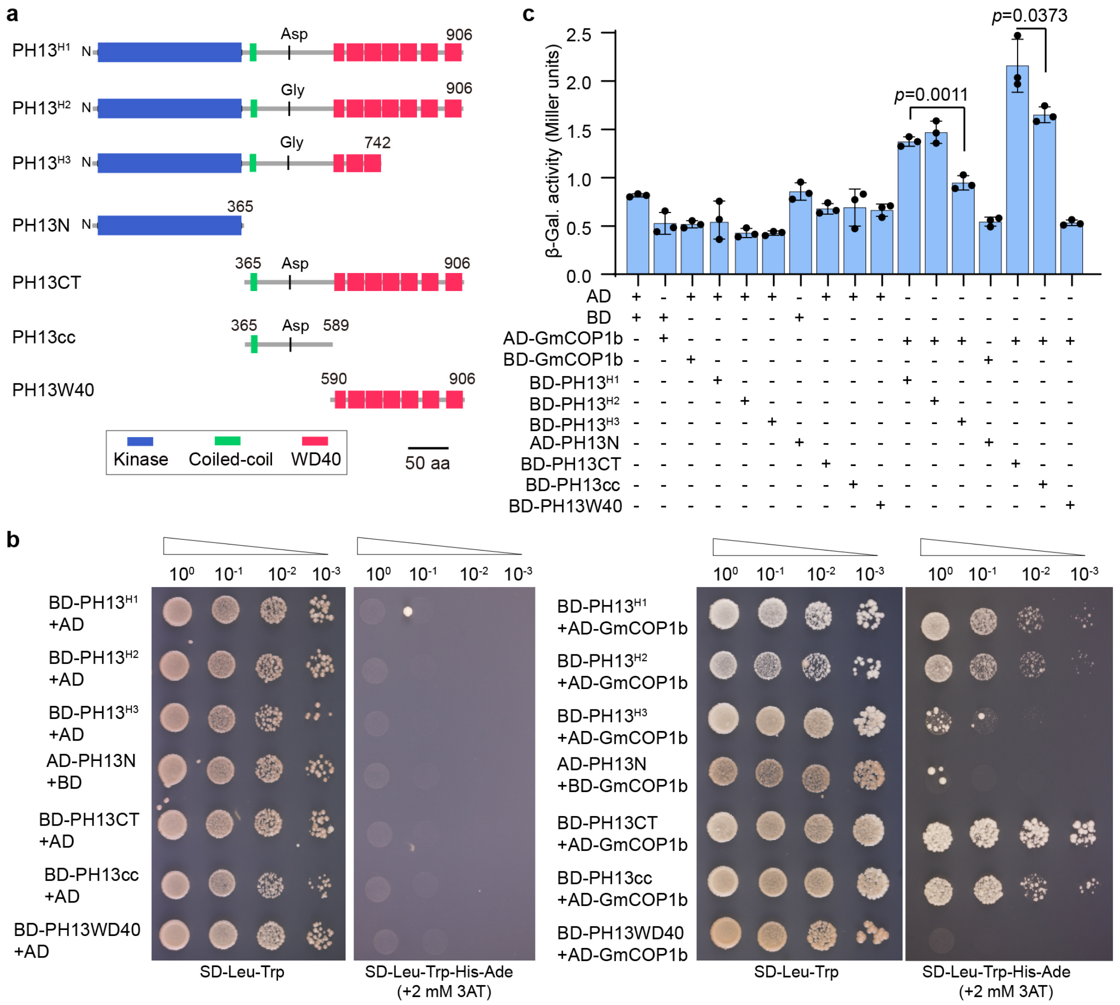

Supplementary Fig. 17: Auxotrophic and β-galactosidase assays showing the interactions between different haplotypes and domains of PH13 with GmCOP1b.

a. Diagram of protein structure of different haplotypes and domains of PH13. **b.** Auxotrophic assays showing the interactions between different haplotypes and domains of PH13 with GmCOP1b. Yeast cells transformed with indicated constructs were selected on -LW (lacking Leu and Trp) or -LWHA (lacking Leu, Trp, His and Ade) medium. **c.** β-galactosidase assays were performed to measure the interaction strength of GmCOP1b with each haplotype and domain of PH13. Data are means ± SD (n = 3), *p* values were calculated by unpaired, two-tailed Student's t-tests. Source data are provided as a Source Data file.

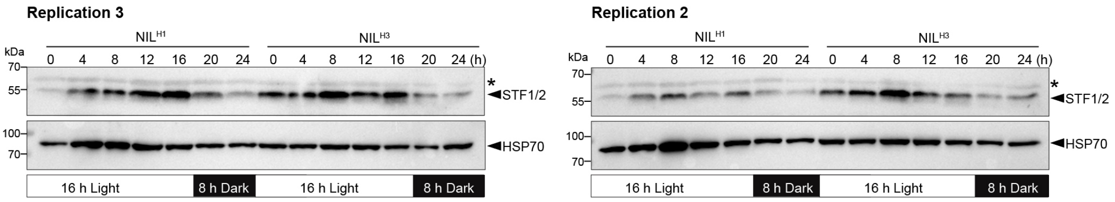

Supplementary Fig. 18: Analysis of STF1/2 abundance in NIL lines.

Biological replication 2 and 3 for STF1/2 abundance in NIL^{H1} and NIL^{H3}. Biological replication 1 is shown in Fig. 4g. The first trifoliate leaves of 15-day-old seedlings grown under long-day condition in a growth chamber were collected at 4-hour intervals. The membrane was probed with the anti-STF1/2 antibody (at a 1:1,000 dilution), stripped, and then probed with the anti-HSP70 antibody (at a 1:10,000 dilution). The asterisk indicates the position of a non-specific band. Source data are provided as a Source Data file.

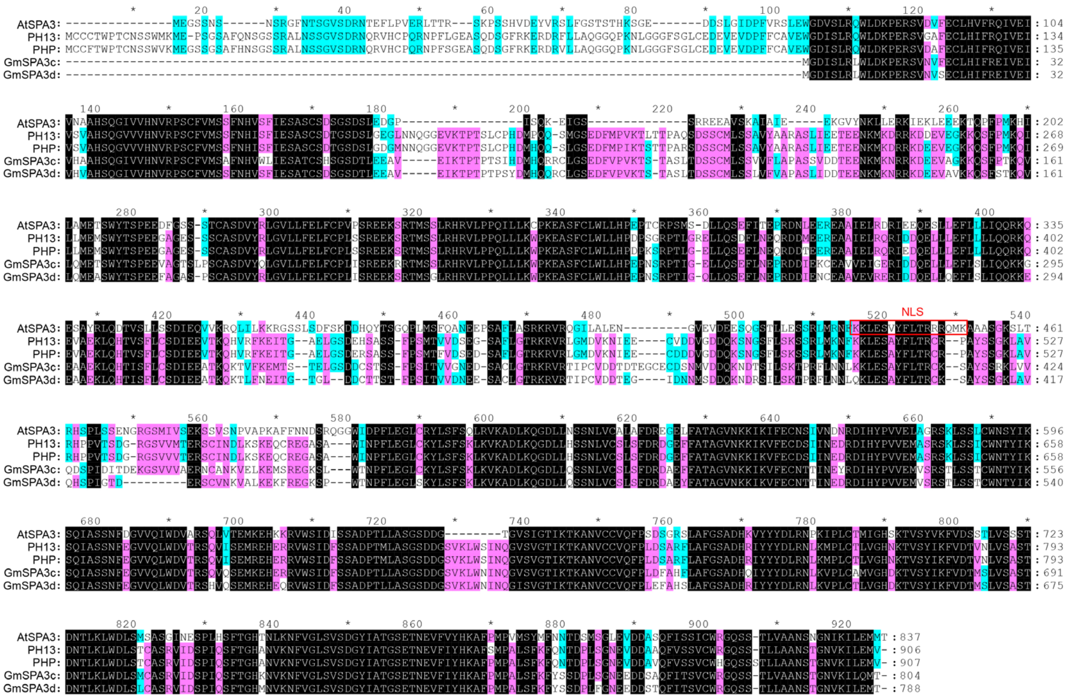

Supplementary Fig. 19: Alignments of SPA3 family proteins in *Arabidopsis* and soybean. The amino acid sequences of AtSPA3, PH13, PHP, GmSPA3c, and GmSPA3d were aligned using ClustalW Multiple alignment in MEGA7 and manually adjusted using GeneDOC software. Red boxes indicate the nuclear localization signals which were identified by the LOCALIZER program (https://localizer.csiro.au/).

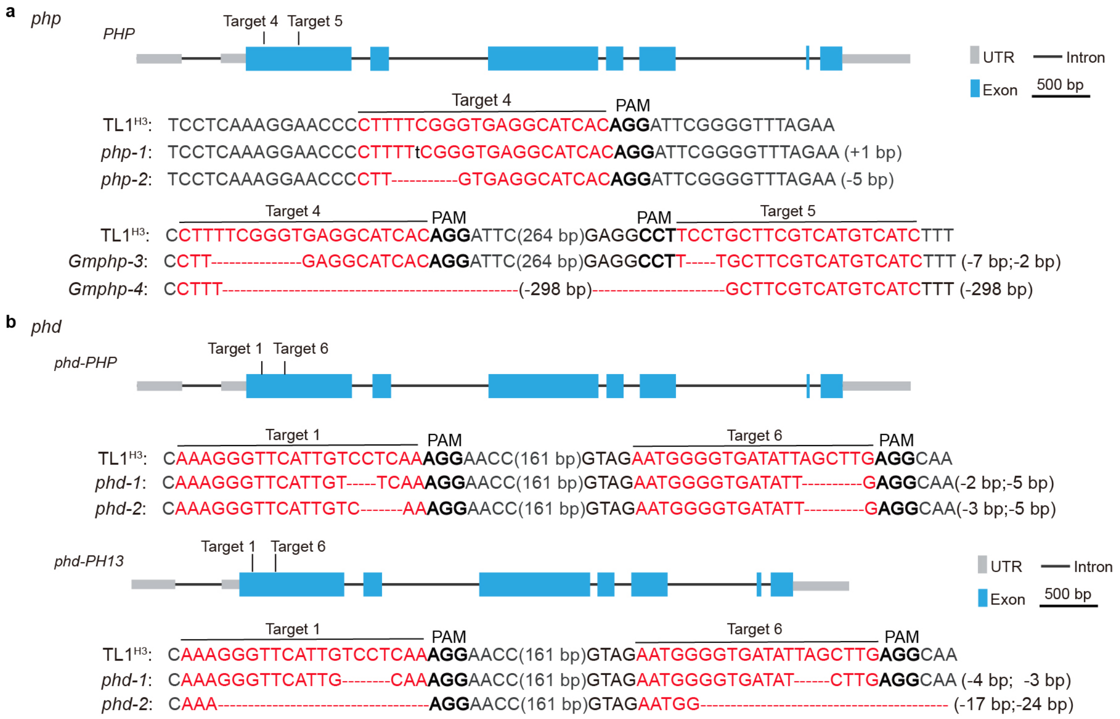

Supplementary Fig. 20: The *phd* and *php* mutant line creation in the CRISPR/Cas9 system. **a and b,** Schematic illustration of the generation of the *php* mutants (**a**) and *phd* double mutants (**b**) in TL1^{H3} background by CRISPR/Cas9 technology. The designed target sites were labeled on the *PHP* and *PH13* genes. The red nucleotides indicate the target sequence, and the bold fonts indicate the protospacer-adjacent motif (PAM). The black letter and dashed lines within the target sites denote nucleotide insertion and deletion, respectively. Data of Sanger sequencing are provided as a Source Data file.

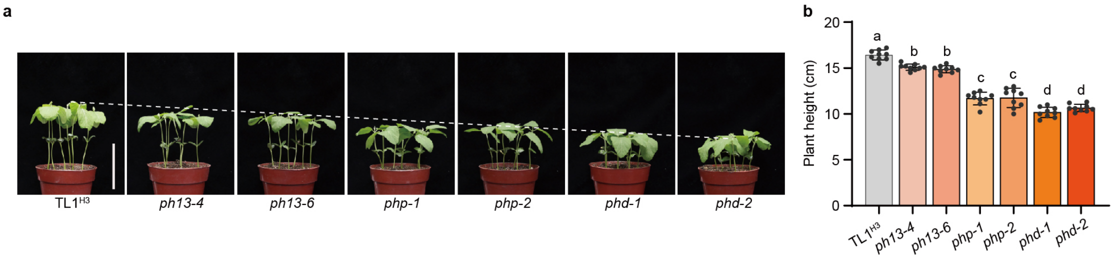

Supplementary Fig. 21: The additive effects of *PH13* and *PHP* genes on stem lengths.

a, Seedling photos of indicated mutant lines and WT plants grown under long day conditions (16 h light/8 h dark) in phytotron. **b,** Statistical analysis of the plant height of the indicated lines as in (**a**). Data are means ± SD (n = 9 biologically independent replicates). Different letters indicate statistically significant differences as determined by one-way ANOVA with two-sided Tukey test at the 0.01 level. Source data are provided as a Source Data file.

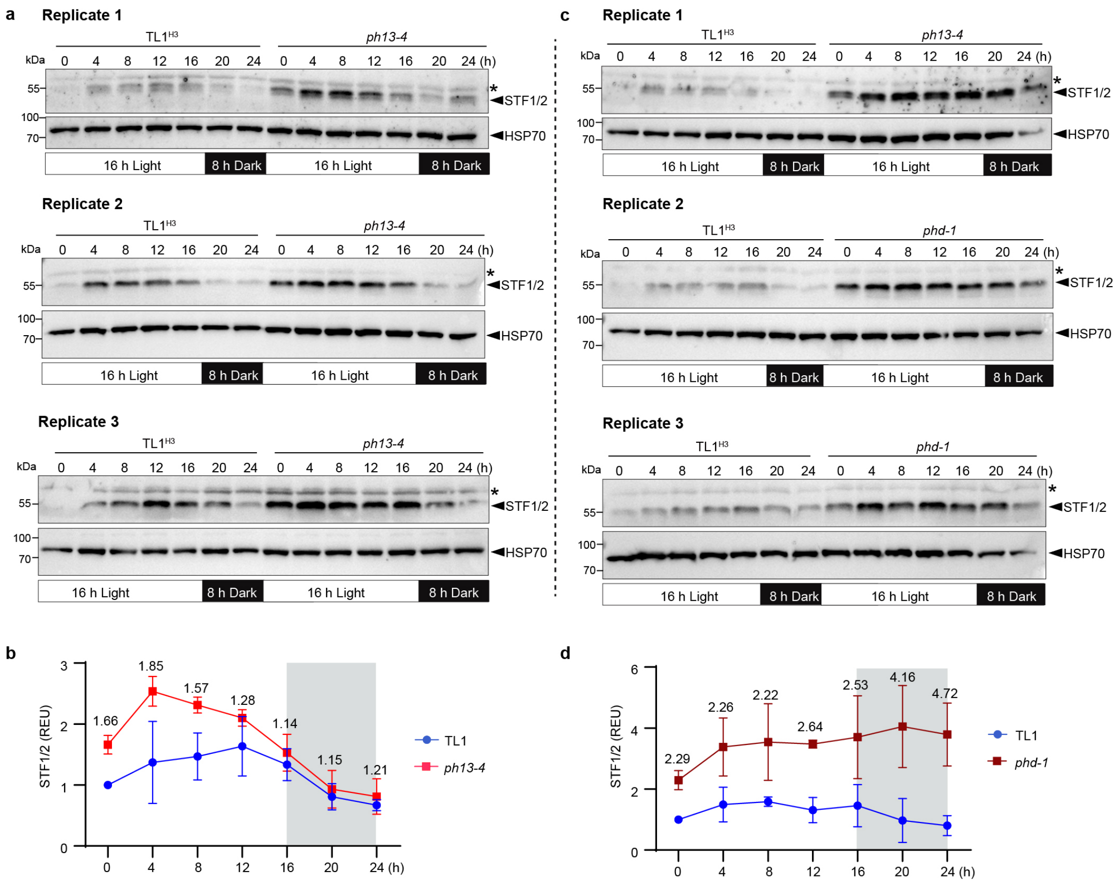

Supplementary Fig. 22: The additive effects of *PH13* and *PHP* genes on STF1/2 abundance. **a and c,** Three biological replicates for immunoblots comparing the diurnal protein levels of STF1/2 in the *ph13-4* (**a**), *phd-1* mutants (**c**), and WT TL1^{H3}. The first trifoliate leaves of 15-day-old seedlings grown under long-day condition in growth chamber were collected every 4 hours. The membrane was probed with the anti-STF1/2 antibody (at a 1:1,000 dilution), stripped, and then probed with the anti-HSP70 antibody (at a 1:10,000 dilution). The asterisk indicates a non-specific band. **b and d,** The relative abundance of STF1/2 protein was calculated using the same formula as in Fig. 4h. Source data are provided as a Source Data file.

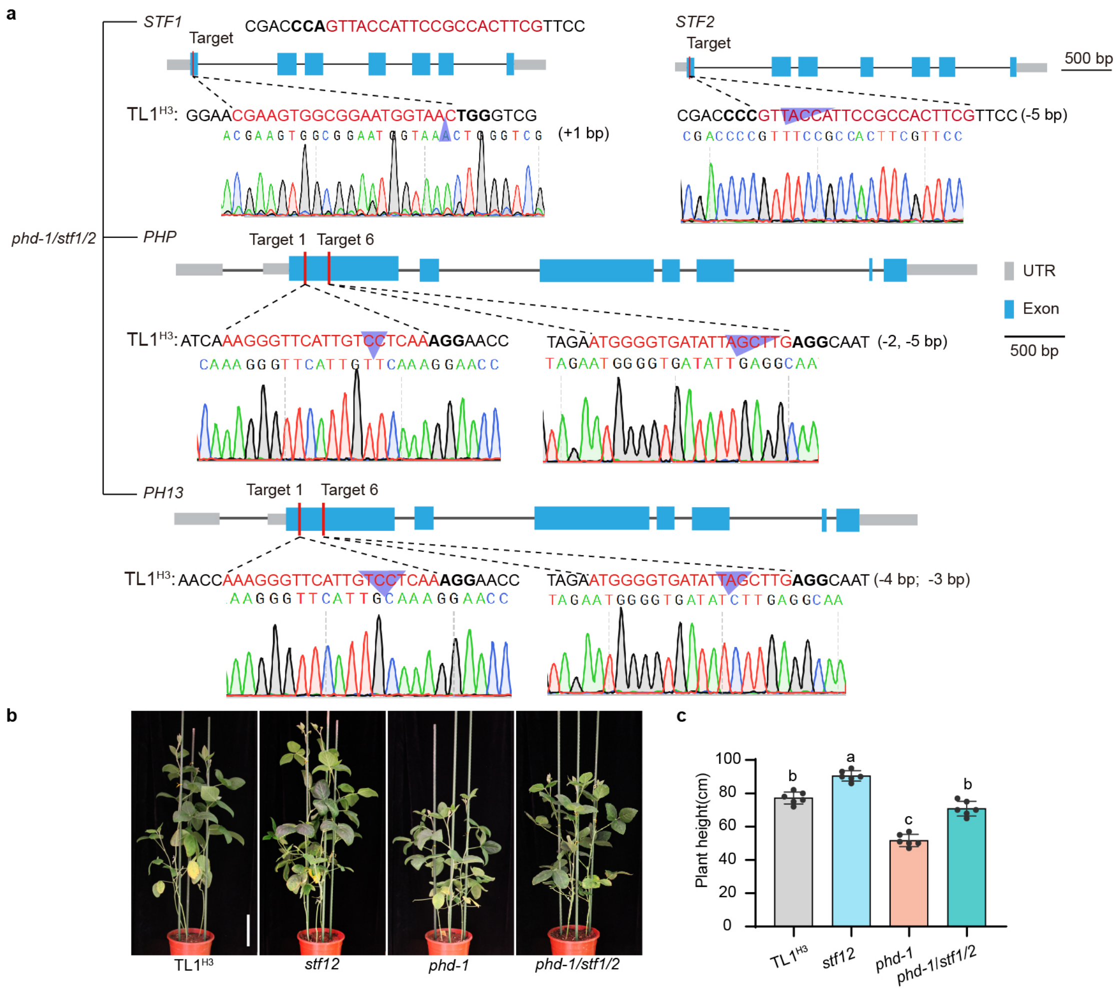

Supplementary Fig. 23: The *stf1/2* mutation partially rescued the dwarf phenotype of the *phd* mutant.

a, Molecular identification of the *phd-1/stf1/2* quadruple mutant by DNA sequencing analysis. The sequence chromatogram shows editing sequences of the CRISPR/Cas9 target site of *PH13*, *PHP*, *STF1*, and *STF2* genes in the quadruple mutant (*phd-1/stf1/2*) and WT TL1^{H3}. The purple triangles within the target sites denote nucleotide insertion and deletion, as determined by Sanger sequencing. **b,** Gross photos of the *stf1/2*, *phd-1*, and *phd-1/stf1/2* mutants and WT TL1^{H3}. Scale bar, 20 cm. **c,** Statistical analysis of the plant height of the indicated lines as in (**b**). Data are means ± SD (n = 6 biologically independent plants). The lowercase letters above the dots indicate significant differences (one-way ANOVA with two-sided Tukey test at the 0.01 level). Source data are provided as a Source Data file.

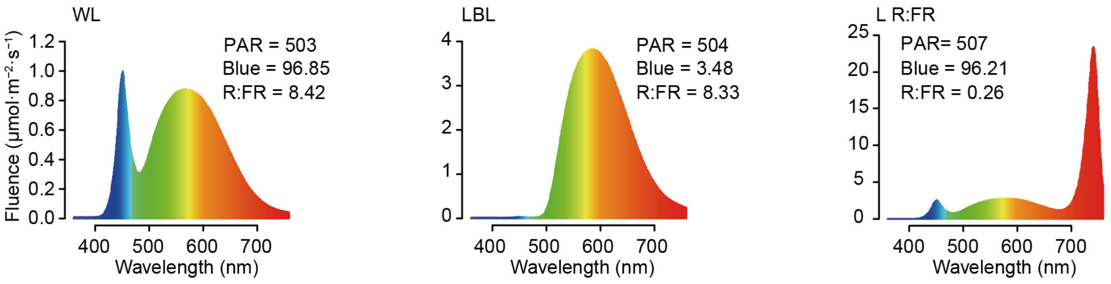

Supplementary Fig. 24: Light spectrum of WL, LBL and L R:FR conditions.

White light (WL), low blue light (LBL), and low red: far-red light (L R:FR) regimes are indicated. The R:FR ratio and blue light fluence rate are shown above each light spectrum. PAR, Photosynthetic Active Radiation. The light regimes are described in Methods-Light Regimes. Source data are provided as a Source Data file.

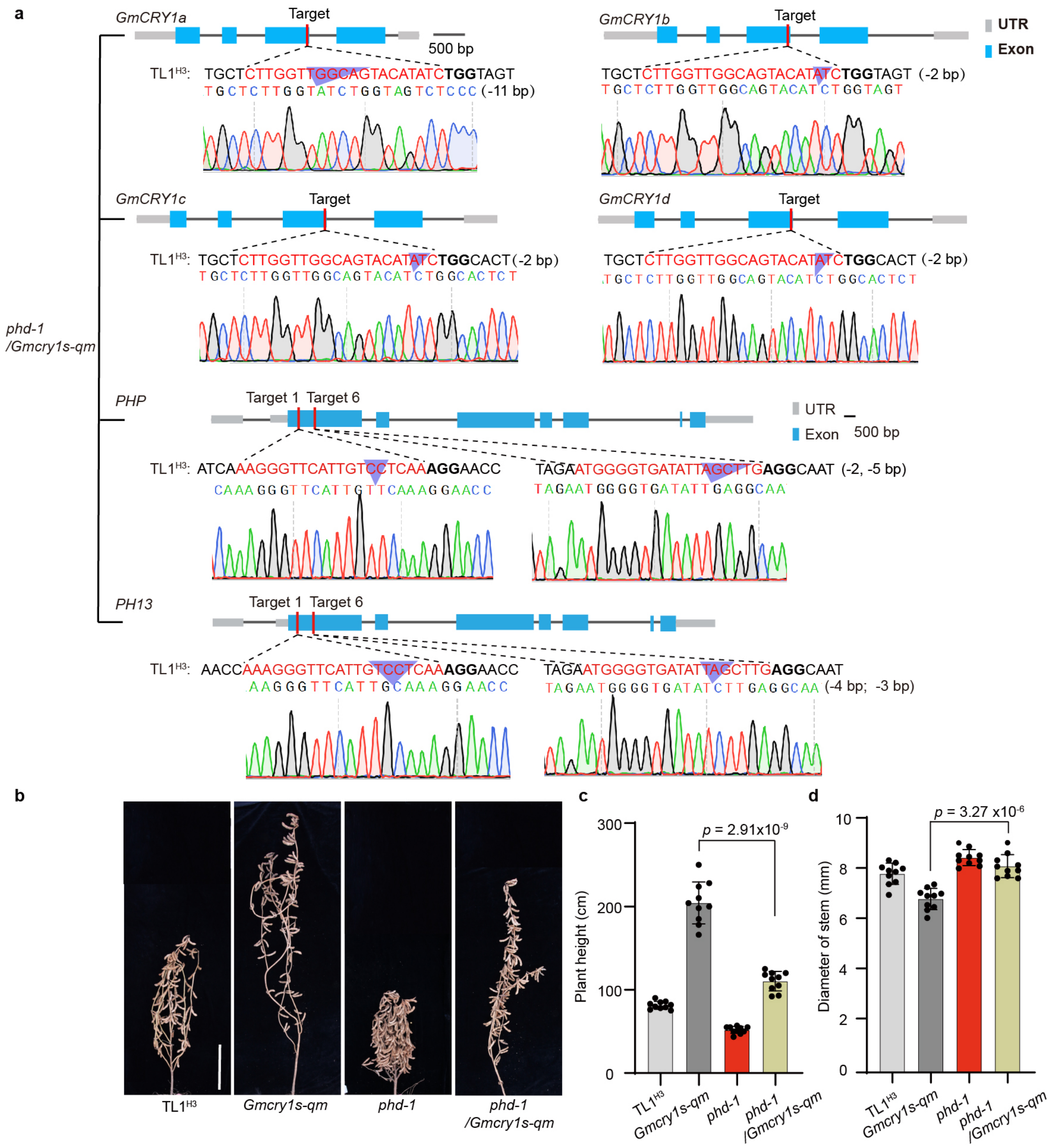

Supplementary Fig. 25: The *phd* mutation partially suppressed the ESE syndrome of the *Gmcry1s-qm* mutant.

a, Molecular identification of the *phd-1/Gmcry1s-qm* mutant by DNA sequencing analysis. The sequence chromatogram shows editing sequences of the CRISPR/Cas9 target site of *GmCRY1a*, *GmCRY1b*, *GmCRY1c*, *GmCRY1d*, *PH13* and *PHP* in the six mutant (*phd-1/Gmcry1s-qm*) and WT TL1^{H3}. The purple triangles within the target sites denote nucleotide insertion and deletion, as determined by Sanger sequencing. **b,** Representative photos of the TL1^{H3}, *Gmcry1s-qm*, *phd-1*, and *phd-1/Gmcry1s-qm* lines grown under Beijing field conditions. Scale bar, 20 cm. **c and d,** Statistical analysis of plant height (**c**) and stem diameter (**d**) of the indicated lines shown in (**b**). Data are means ± SD (n = 10 biologically independent plants) with *p* values calculated by unpaired, two-tailed Student's t-tests. Source Data are provided as a Source Data file.

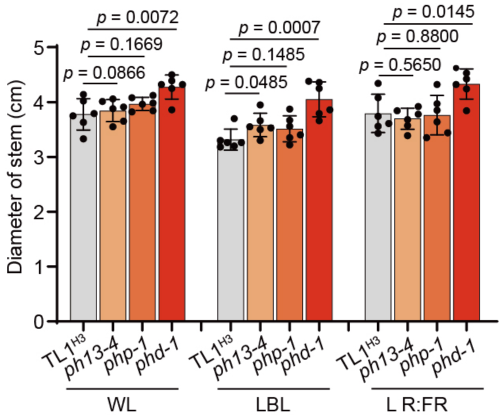

Supplementary Fig. 26: *PH13* and *PHP* redundantly regulate stem diameter.

Statistical analysis of stem diameter of the $TL1^{H3}$, *ph13*, *php*, and *phd* lines shown in Fig. 5b. Data are means ± SD (n = 6 biologically independent plants) with *p* values calculated by unpaired, two-tailed Student's t-tests. Source data are provided as a Source Data file.

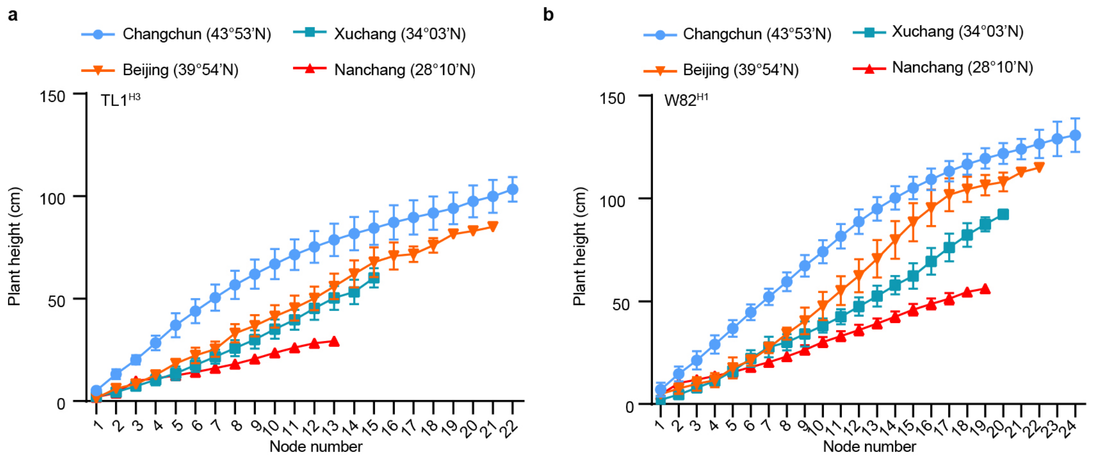

Supplementary Fig. 27: Changes in plant height and internode length of TL1^{H3} and W82^{H1} cultivars in different latitudes.

The curves showing stem length at each node of the TL1^{H3} cultivar (**a**) and W82^{H1} cultivar (**b**) in Changchun (125°19′E, 43°53′N), Beijing (116°23′E, 39°54′N), Xuchang (104°31′E, 34°10′N) and Nanchang (115°27′E, 28°10′N) fields in 2022. The data were collected before maturity. Data are means ± SD (n = 10 biologically independent plants). Source data are provided as a Source Data file.

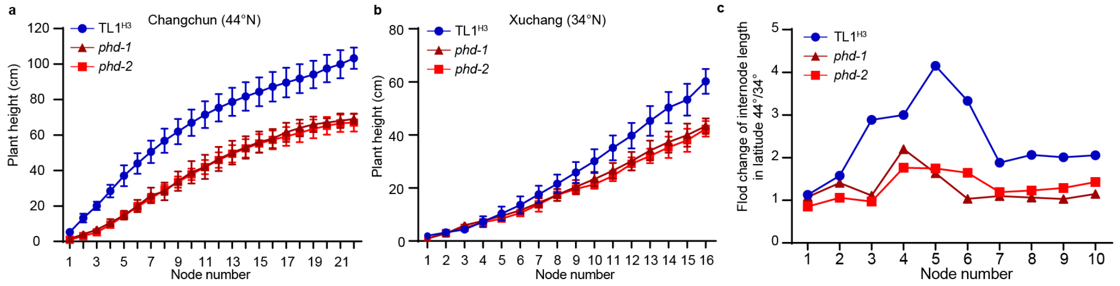

Supplementary Fig. 28: Comparison of the internode length of TL1^{H3} and *phd* mutant lines in different latitudes.

a-b, Curves showing plant height at each node of indicated lines grown in Changchun (125°19′E, 43°53′N) (**a**) and Xuchang (104°31′E, 34°10′N) (**b**). **c,** Fold change in internode length in Changchun relative to that in Xuchang as in panels (**a**) and (**b**). Data are means ± SD (n = 10 biologically independent plants) collected at vegetation stage 8 (V8). Source data are provided as a Source Data file.

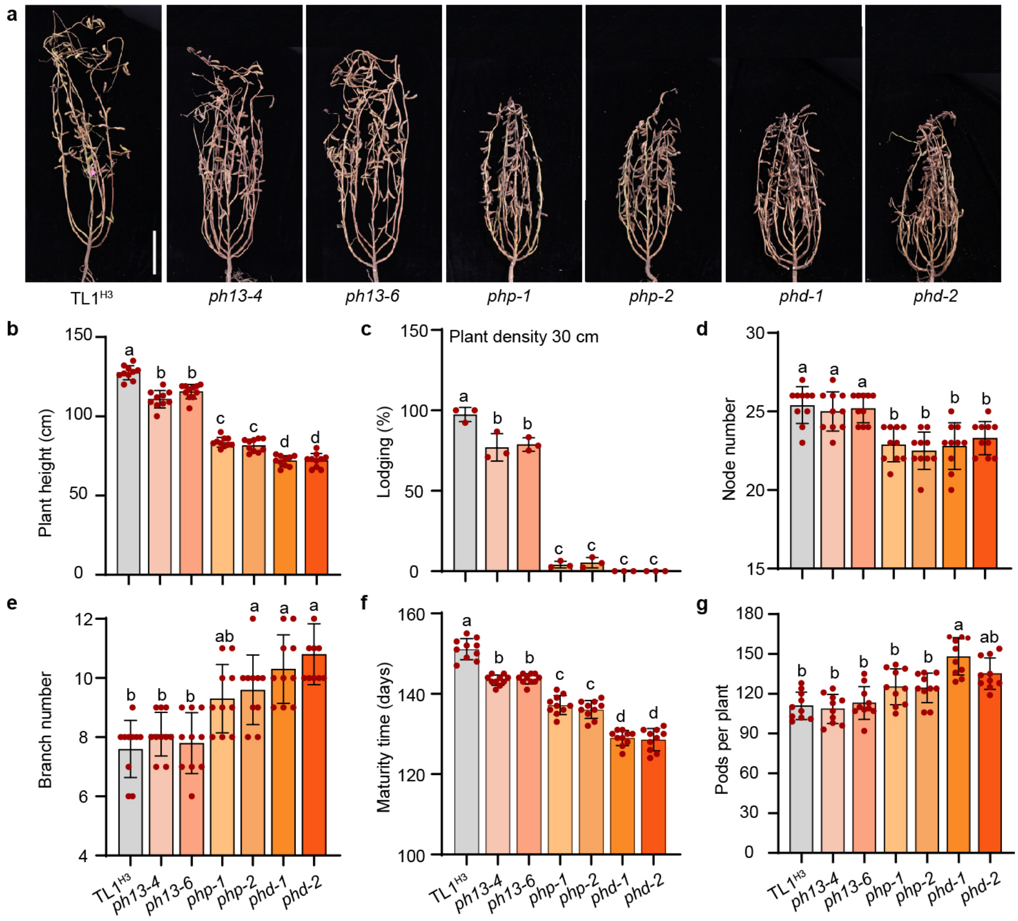

Supplementary Fig. 29: The *phd* mutation enhanced the adaptability of soybean at high latitude.

a, Photos of the WT TL1 and *ph13*, *php*, and *phd* mutants exhibiting a range of dwarf phenotype in Changchun field trails. Scale bar, 20 cm. **b-g,** Statistical analysis of the plant height (**b**), lodging rate (**c**), node number (**d**), branch number (**e**), maturity time (**f**), and pods per plant (**g**) of the indicated lines as in (**a**). Data are means ± SD (n = 10 biologically independent plants). Lowercase letters above the dots indicate significant differences (one-way ANOVA with two-sided Tukey test at the 0.01 level). Source data are provided as a Source Data file.

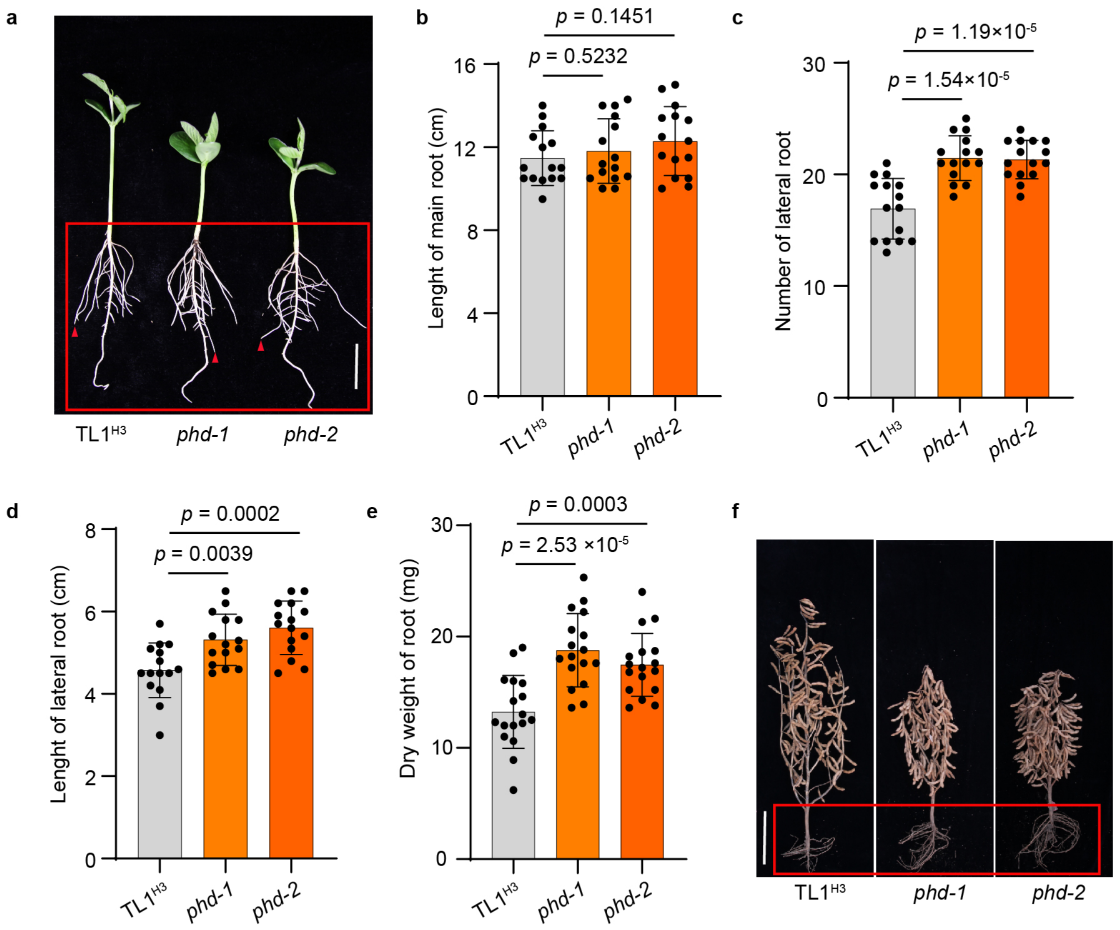

Supplementary Fig. 30: Effects of the *phd* mutation on root architecture of soybean.

a, Seedling phenotypes of *phd-1* mutants and WT TL1^{H3} grown under long day conditions (16 h light/8 h dark) in phytotron. The red arrow represents the position of the longest lateral root. Scale bar, 2 cm. **b-e,** Statistical analysis of the length of main root, number of lateral root, length of lateral root, and dry weight of root of the indicated lines as in (**a**). Data are means ± SD (n = 15 biologically independent plants) with *p* values calculated by unpaired, two-tailed Student's t-tests. **f,** Representative images of the indicated lines grown under Beijing field conditions in 2021. Scale bar, 20 cm. Source data are provided as a Source Data file.

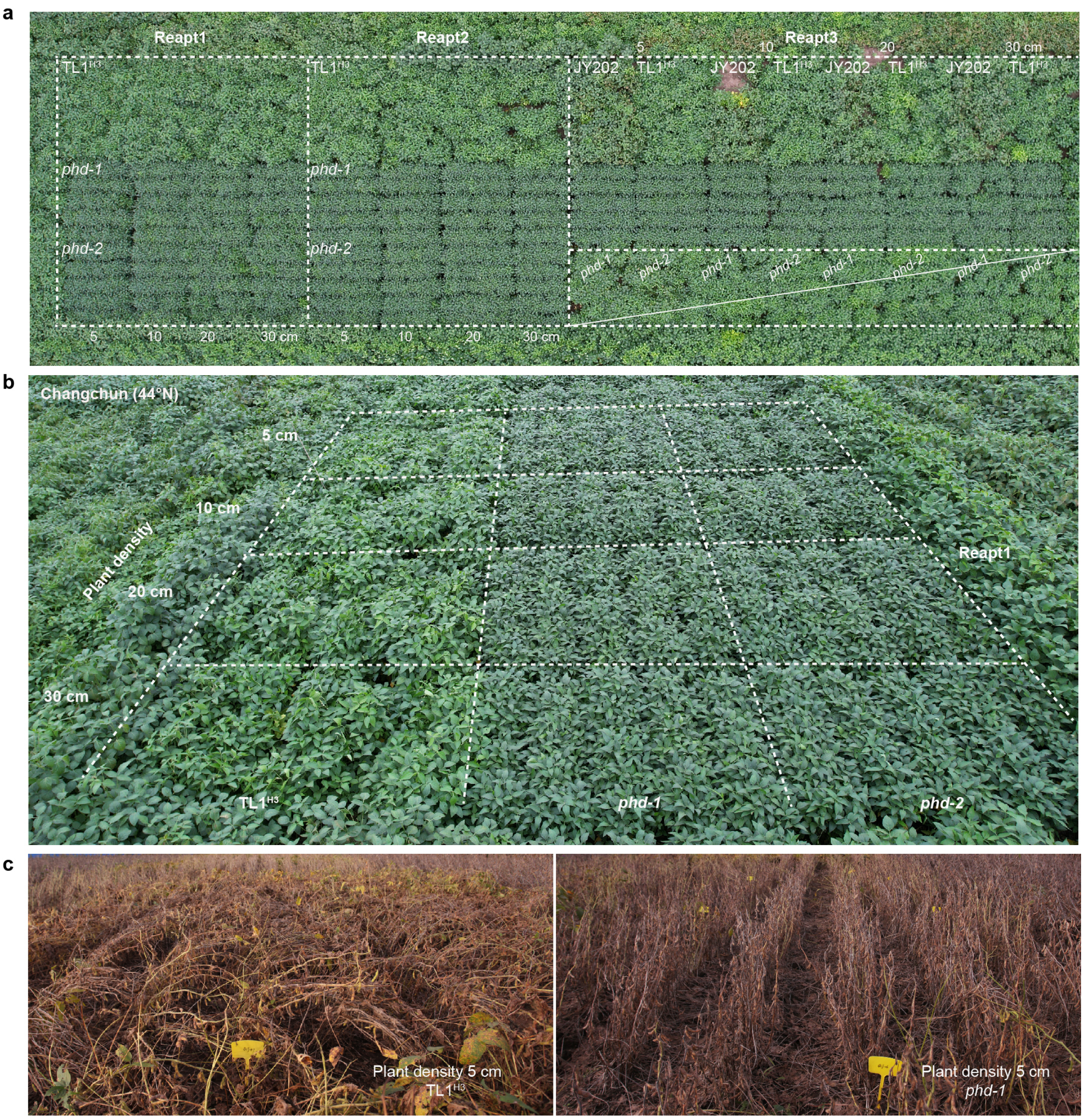

Supplementary Fig. 31: The *phd* mutants were lodging resistant under different planting density at high latitude.

a-c, Photos of the WT TL1^{H3} and *phd* mutants at the vegetative stage (**a** and **b**) and maturity stage (**c**) under increasing densities in the Changchun field.

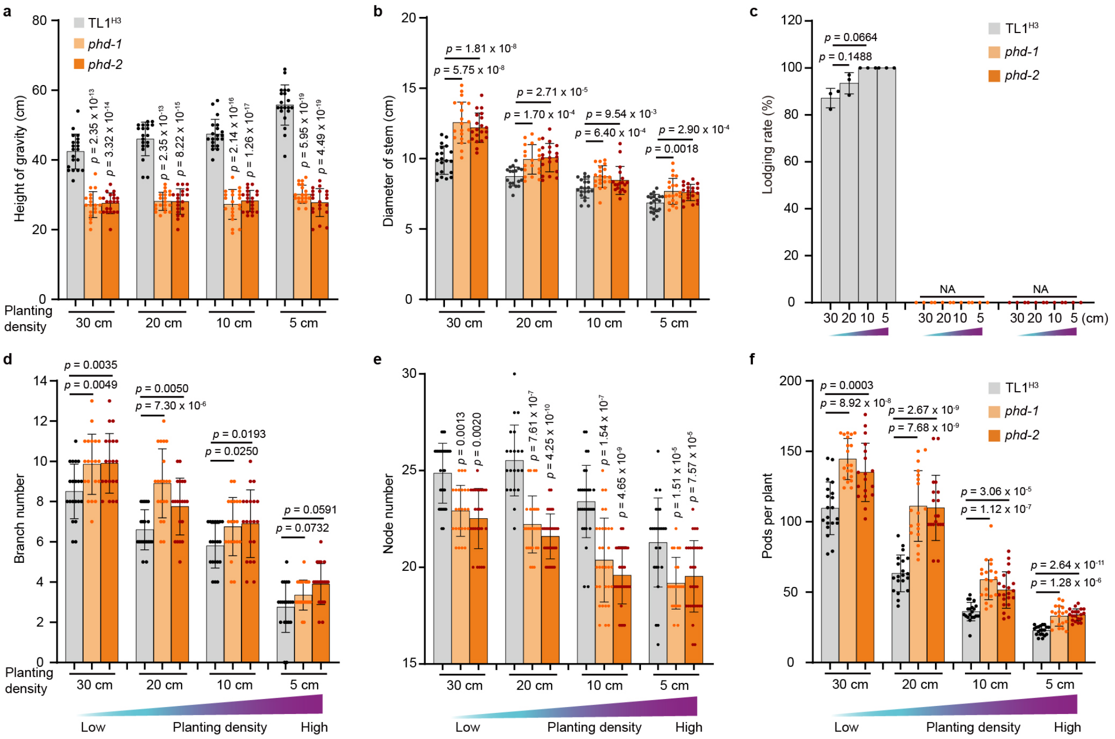

Supplementary Fig. 32: Statistical analysis of agronomic traits of the *phd* mutants and WT TL1^{H3} under different planting density.

a-f, Statistical analysis of height of the center gravity point (**a**), diameter of stem (**b**), lodging rate (**c**), branch number (**d**), node number (**e**), and pods per plant (**f**) of the *phd* mutants and TL1 under different planting density. Data are shown as mean values ± SD (n = 20 biologically independent plants; except for n = 3 biologically independent replicates for **c**). *p* values were calculated by unpaired, two-tailed Student's t-tests. Source data are provided as a Source Data file.

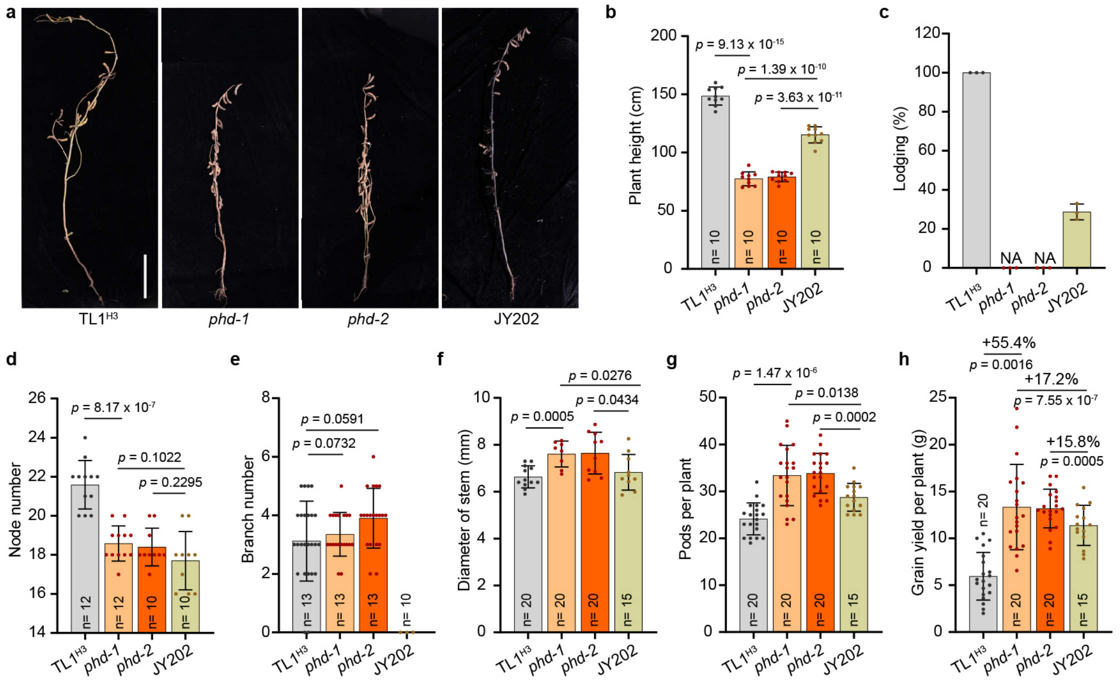

Supplementary Fig. 33: Comparison of the agronomic traits of the *phd* mutants, WT TL1^{H3}, and a local elite cultivar JY202.

a, Photos of the indicated lines grown under 5 cm planting density in Changchun field in 2021. Scale bar, 20 cm. **b-h,** Statistical analysis of plant height (**b**), the lodging rate (**c**), node number (**d**), branch number (**e**), diameter of stem (**f**), pods per plant (**g**), and grain yield per plant (**h**) in the field traits. Data are mean values ± SD (n > 10 biologically independent plants, except for c, n = 3 biologically independent field plots). *p* values were calculated by unpaired, two-tailed Student's t-tests. Source data are provided as a Source Data file.

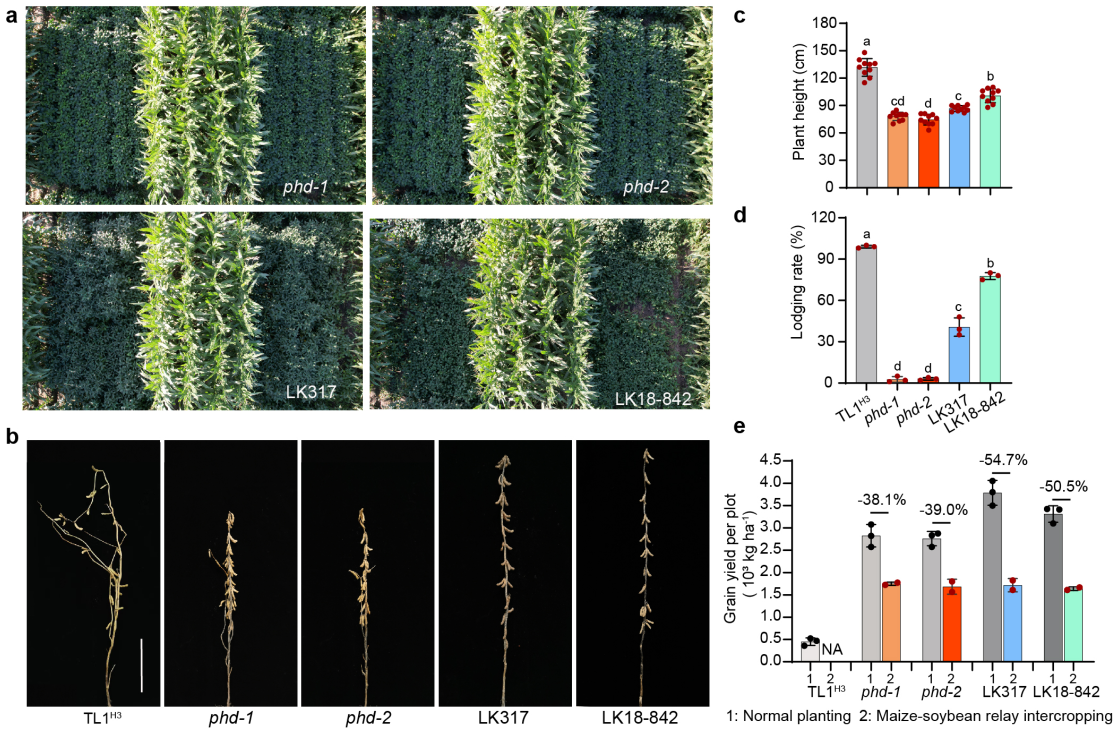

Supplementary Fig. 34: Performance of the *phd* mutants under maize-soybean relay intercropping conditions.

a, Photos of the *phd* mutant lines and two local commercial varieties LK317 and LK18-842 at vegetative stage under maize-soybean intercropping conditions in Harbin (45°7'N, 127°5'E). **b,** Gross photos of indicate lines as in (**a**) at mature stage. Scale bar, 20 cm. **c-e,** Statistical analysis of plant height (**c**) and lodging rate (**d**). Data are means ± SD (n = 10 biologically independent plants for **c**; n = 3 biologically independent field plots for **d**). The lowercase letters above the dots indicate significant differences (one-way ANOVA with two-sided Tukey test at the 0.01 level). **e,** Grain yield per plot of at least two replicates. The values above the bars indicate the percentage of yield reduction under maize-soybean intercropping conditions relative to that under soybean monoculture conditions. Source data are provided as a Source Data file.
