# Supplementary material for: PH13 improves soybean shade traits and enhances yield for high-density planting at high latitudes
Source: Nat Commun. 2023 Oct 26;14:6813. doi: 10.1038/s41467-023-42608-5 (PMC10603158; doi:10.1038/s41467-023-42608-5)
Supplement: Supplementary file 3 — Description of Additional Supplementary Files [file 41467_2023_42608_MOESM3_ESM.pdf]

Description of Additional Supplementary Files

Supplementary Data 1-8:

Supplementary Data 1. Accession of GWAS and TWAS in this study.

Supplementary Data 2. Overlap between our GWAS loci and reported GWAS QTLs or gene.

Supplementary Data 3. List of plant height loci identified by TWAS.

Supplementary Data 4. The amino acid sequence of SPAs and its homologous proteins.

Supplementary Data 5. The conserved domains of insertion sequence were analyzed using the Blast N method in NCBI.

Supplementary Data 6. The sequence of *Ty1/Copia*-like retrotransposon.

Supplementary Data 7. The haplotype, plant height and geographic information of soybean accessions in this study.

Supplementary Data 8. The primer in this study.
