# Supplementary material for: PH13 improves soybean shade traits and enhances yield for high-density planting at high latitudes
Source: Nat Commun. 2023 Oct 26;14:6813. doi: 10.1038/s41467-023-42608-5 (PMC10603158; doi:10.1038/s41467-023-42608-5)
Supplement: Supplementary file 12 — Reporting Summary [file 41467_2023_42608_MOESM12_ESM.pdf]

nature portfolio

Corresponding author(s): Bin Lin

Last updated by author(s): Sep 21, 2023

Reporting Summary

Nature Portfolio wishes to improve the reproducibility of the work that we publish. This form provides structure for consistency and transparency in reporting. For further information on Nature Portfolio policies, see our Editorial Policies and the Editorial Policy Checklist.

Statistics

For all statistical analyses, confirm that the following items are present in the figure legend, table legend, main text, or Methods section.

n/a	Confirmed	
☐	☒	The exact sample size (*n*) for each experimental group/condition, given as a discrete number and unit of measurement
☐	☒	A statement on whether measurements were taken from distinct samples or whether the same sample was measured repeatedly
☐	☒	The statistical test(s) used AND whether they are one- or two-sided<br>*Only common tests should be described solely by name; describe more complex techniques in the Methods section.*
☒	☐	A description of all covariates tested
☒	☐	A description of any assumptions or corrections, such as tests of normality and adjustment for multiple comparisons
☐	☒	A full description of the statistical parameters including central tendency (e.g. means) or other basic estimates (e.g. regression coefficient) AND variation (e.g. standard deviation) or associated estimates of uncertainty (e.g. confidence intervals)
☐	☒	For null hypothesis testing, the test statistic (e.g. *F*, *t*, *r*) with confidence intervals, effect sizes, degrees of freedom and *P* value noted<br>*Give P values as exact values whenever suitable.*
☒	☐	For Bayesian analysis, information on the choice of priors and Markov chain Monte Carlo settings
☒	☐	For hierarchical and complex designs, identification of the appropriate level for tests and full reporting of outcomes
☒	☐	Estimates of effect sizes (e.g. Cohen's *d*, Pearson's *r*), indicating how they were calculated

Our web collection on statistics for biologists contains articles on many of the points above.

Software and code

Policy information about availability of computer code

Data collection

The phenotypes of plant materials and yeast were acquired using the Canon EOS 5D Mark II.
The amino acid sequences were aligned by ClustalW Multiple alignments in DNAMAN V6.
The subcellular localization of three PH13 haplotypes were acquired by confocal microscopy: Zeiss LSM700.
The immunoblot analysis were acquired by Tanon-5200.
The light spectrum were acquired by HiPoint HR-350 and were draw by R software (Packages: readxl).
The photos of different planting density and intercropping were acquired by DJI Mavic Air 2s.

Data analysis

Phylogenetic analysis of PH13 homologs was analyzed using the MEGA7.0.26 software.
Real-time expression data were analyzed using the Biometra Tone 96 G.
The graph of geographical distribution of three PH13 haplotypes were draw by R software (Packages:sf, ggspatial, ggplot2, patchwork, RColorBrewer, cowplot, tidyverse, colorspace, openxlsx) and its cthe relevant code was provided in Figshare.
Quantitation of protein abundance were analyzed using ImageJ V2.3.0.
Data analysis were performed using Graphpad Prism V9.4.1 and Microsoft Excel.

For manuscripts utilizing custom algorithms or software that are central to the research but not yet described in published literature, software must be made available to editors and reviewers. We strongly encourage code deposition in a community repository (e.g. GitHub). See the Nature Portfolio guidelines for submitting code & software for further information.

Data

Policy information about availability of data

All manuscripts must include a data availability statement. This statement should provide the following information, where applicable:

- Accession codes, unique identifiers, or web links for publicly available datasets
- A description of any restrictions on data availability
- For clinical datasets or third party data, please ensure that the statement adheres to our policy

All data supporting the findings of this work are available within the paper and its Supplementary Information files. The datasets and plant materials generated and analyzed during the current study are available from the corresponding author upon request. The genotyped data had the DNA sequence reads deposited as PRJNA681974 (Li et al., 2023) in the Sequence Read Archive database of NCBI, and expression data had the RNA-Seq sequence reads deposited as PRJCA014188 in Genome Sequence Archive (GSA) database of BIG Data Center. All soybean accessions were analyzed in this study using GWAS, TWAS and the analysis of geographical distribution were listed in Supplementary Data 1 and 2. All protein accession numbers listed in Supplementary Data 3 are publicly available at NCBI Genebank (https://www.ncbi.nlm.nih.gov/). All gene sequences may be obtained from the Phytozome database (https://phytozome-next.jgi.doe.gov/info/Gmax_Wm82_a2_v1), the hyperlinks of all accession codes are listed in Supplementary Table 4.

Research involving human participants, their data, or biological material

Policy information about studies with human participants or human data. See also policy information about sex, gender (identity/presentation), and sexual orientation and race, ethnicity and racism.

Reporting on sex and gender	N/A
Reporting on race, ethnicity, or other socially relevant groupings	N/A
Population characteristics	N/A
Recruitment	N/A
Ethics oversight	N/A

Note that full information on the approval of the study protocol must also be provided in the manuscript.

Field-specific reporting

Please select the one below that is the best fit for your research. If you are not sure, read the appropriate sections before making your selection.

☒ Life sciences ☐ Behavioural & social sciences ☐ Ecological, evolutionary & environmental sciences

For a reference copy of the document with all sections, see nature.com/documents/nr-reporting-summary-flat.pdf

Life sciences study design

All studies must disclose on these points even when the disclosure is negative.

Sample size	Sample sizes were determined based on preliminary data demonstrating statistically significant differences for each specific assay.
Data exclusions	No data were excluded from the analysis.
Replication	The replication of experiments in this study were described in the legend.
Randomization	Soybean plants grown in the plant growth chambers or natural field conditions were randomized along with all required controls. The samples tested were randomized and replicated in all experiments.
Blinding	Blinding was not relevant as all processing methods were done through available software with consistent parameters utilized across all soybean plants and required controls.

Reporting for specific materials, systems and methods

We require information from authors about some types of materials, experimental systems and methods used in many studies. Here, indicate whether each material, system or method listed is relevant to your study. If you are not sure if a list item applies to your research, read the appropriate section before selecting a response.

Materials & experimental systems

n/a	Involved in the study	
☐	☒	Antibodies
☒	☐	Eukaryotic cell lines
☒	☐	Palaeontology and archaeology
☒	☐	Animals and other organisms
☒	☐	Clinical data
☒	☐	Dual use research of concern
☐	☒	Plants

Methods

n/a	Involved in the study	
☒	☐	ChIP-seq
☒	☐	Flow cytometry
☒	☐	MRI-based neuroimaging

Antibodies

Antibodies used	Commercial antibodies: anti-GFP (MBL, 598, 1:2500 dilution), anti-Flag (Abmart, M20008L, 1:2500 dilution), anti-HSP (BPI, AbM51099-31-PU, 1:10000 dilution).
Validation	anti-GFP (http://www.mbl-chinawide.cn/uploads/pdf/598-v13.pdf); anti-FLAG (http://www.ab-mart.com.cn/upload/20211217182008xz.pdf); anti-HSP (http://www.proteomics.org.cn/product/202.html).

Dual use research of concern

Policy information about dual use research of concern

Hazards

Could the accidental, deliberate or reckless misuse of agents or technologies generated in the work, or the application of information presented in the manuscript, pose a threat to:

No	Yes	
☒	☐	Public health
☒	☐	National security
☒	☐	Crops and/or livestock
☒	☐	Ecosystems
☒	☐	Any other significant area

Experiments of concern

Does the work involve any of these experiments of concern:

No	Yes	
☒	☐	Demonstrate how to render a vaccine ineffective
☒	☐	Confer resistance to therapeutically useful antibiotics or antiviral agents
☒	☐	Enhance the virulence of a pathogen or render a nonpathogen virulent
☒	☐	Increase transmissibility of a pathogen
☒	☐	Alter the host range of a pathogen
☒	☐	Enable evasion of diagnostic/detection modalities
☒	☐	Enable the weaponization of a biological agent or toxin
☒	☐	Any other potentially harmful combination of experiments and agents
